# Pathogenesis of Cluster 1 Duck Tembusu Virus in Ducks Reveals the Impact of Viral Genotype on Pathogenicity and Disease Severity

**DOI:** 10.1155/2023/9239953

**Published:** 2023-11-28

**Authors:** Wikanda Tunterak, Kanana Rungprasert, Suwarak Wannaratana, Nichapat Yurayart, Duangduean Prakairungnamthip, Patchareeporn Ninvilai, Benchaphorn Limcharoen, Teerawut Nedumpun, Rodolphe Hamel, Wijit Banlunara, Aunyaratana Thontiravong

**Affiliations:** ^1^Department of Veterinary Microbiology, Faculty of Veterinary Science, Chulalongkorn University, Bangkok, Thailand; ^2^Faculty of Veterinary Medicine, Rajamangala University of Technology Tawan-Ok, Chonburi, Thailand; ^3^Animal Vector-Borne Disease Research Unit, Veterinary Parasitology Unit, Department of Pathology, Faculty of Veterinary Science, Chulalongkorn University, Bangkok, Thailand; ^4^Avian Veterinary Services, CPF (Thailand) Public Company Limited, Bangkok, Thailand; ^5^Department of Anatomy, Faculty of Veterinary Science, Chulalongkorn University, Bangkok, Thailand; ^6^MIVEGEC, Univ Montpellier, CNRS, IRD, Montpellier, France; ^7^Department of Clinical Microbiology and Applied Technology, Faculty of Medical Technology, Mahidol University, Nakhon Pathom, Thailand; ^8^Viral Vector Joint Unit and Join Laboratory, Mahidol University, Nakhon Pathom, Thailand; ^9^Department of Pathology, Faculty of Veterinary Science, Chulalongkorn University, Bangkok, Thailand; ^10^Center of Excellence for Emerging and Re-emerging Infectious Diseases in Animals (CUEIDAs), Faculty of Veterinary Science, Chulalongkorn University, Bangkok, Thailand; ^11^Center of Excellence of Systems Microbiology, Faculty of Medicine, Chulalongkorn University, Bangkok, Thailand

## Abstract

Duck Tembusu virus (DTMUV), an emerging avian pathogenic flavivirus, causes severe neurological disorders and acute egg drop syndrome in ducks. Currently, several clusters of DTMUV, including clusters 1, 2, and 3, have been identified and caused outbreaks in Asia. However, most of the DTMUV pathogenesis evaluation has mainly focused on cluster 2, while limited information is available on the pathogenesis of other DTMUV clusters, particularly cluster 1. In this study, the pathogenesis of a cluster 1 DTMUV was investigated in Cherry Valley ducks and compared to our previously reported cluster 2.1 DTMUV. Our results demonstrated that cluster 1 DTMUV was generally less pathogenic than cluster 2.1 DTMUV in ducks as evidenced by slower body weight loss, lower morbidity and mortality rates, and milder pathological changes. Concordantly, delayed viremia, reduced viral loads in blood and tissues, and shorter shedding period with lower viral loads were also observed in cluster 1 DTMUV inoculated ducks compared with those reported in cluster 2.1 DTMUV. In addition, we also found that cluster 1 DTMUV exhibited significant antigenic difference compared to cluster 2.1 DTMUV. Altogether, our findings suggest distinct pathogenicity and antigenicity between cluster 1 and 2.1 DTMUVs in ducks, highlighting the potential association between DTMUV genotype and pathogenicity/disease severity. This study enhances our understanding of DTMUV pathogenesis in ducks and provides useful information for the design and development of effective DTMUV vaccines.

## 1. Introduction

Duck Tembusu virus (DTMUV) is an emerging mosquito-borne flavivirus that causes severe neurological disorders and acute egg drop syndrome in ducks and some other avian species, including geese and chickens [1]. Currently, DTMUV is widely spread and becomes one of the most economically important disease of poultry in Asia [2–5]. To establish effective control and prevention strategies, a better understanding of the pathogenesis of this emerging virus is crucial.

DTMUV is classified as a new genotype of Tembusu virus (TMUV), which belongs to the genus *Flavivirus* of the family *Flaviviridae* [6, 7]. Like other flaviviruses, DTMUV genome consists of a positive-sense single-stranded RNA, which encodes three structural proteins (capsid (C), premembrane protein (prM), and envelope (E)) and seven nonstructural proteins (NS1, NS2A, NS2B, NS3, NS4A, NS4B, and NS5) [1]. At present, DTMUV is genetically classified into three distinct clusters, including cluster 1, cluster 2 (2.1 and 2.2), and cluster 3 [2]. All three clusters were found to be circulated in Asia with different geographical distributions [2]. Interestingly, our previous study revealed a marked genetic divergence among these three different clusters of DTMUV [2]. Whether these genetic changes contributed to the antigenicity, pathogenicity, and disease severity, however, remains largely unknown.

Several studies consistently showed that DTMUV generally causes retarded growth and severe neurological syndromes in young ducks and decreased egg production in laying ducks [8–10]. However, the severity of the disease varies greatly from mild to severe clinical symptoms depending on several factors, including the age of duck, the strain of virus, the route of infection, and the virus infectivity titer [10–13]. Besides the duck age, the viral strain/genotype is likely to be a key factor affecting the virulence and pathogenicity of DTMUV [12–14]. However, the pathogenesis evaluation of DTMUV has been mainly restricted to cluster 2. Limited information is available on the pathogenesis of other DTMUV clusters, particularly cluster 1, which was circulated in some Asian countries, including Malaysia and Thailand. Here, the pathogenesis of a cluster 1 DTMUV was investigated in Cherry Valley ducks and compared to our previously reported cluster 2.1 DTMUV [10].

## 2. Materials and Methods

### 2.1. Virus

DTMUV strain DK/TH/CU-DTMUV2007 (GenBank accession number MF621927) was used for pathogenesis assessment in this study [15]. This virus was originally obtained from ducks infected with DTMUV in Thailand and belongs to the DTMUV cluster 1 [2, 15]. The propagation of DK/TH/CU-DTMUV2007 was carried out in 9-day-old embryonated duck eggs, as described previously [7]. The virus was subsequently collected, clarified through centrifugation, and the 50% embryo lethal dose (ELD_50_) was determined using the Reed and Muench's [16] method. All viral handling was conducted in a BSL-2 containment facility.

### 2.2. Animal Study

The pathogenesis assessment of cluster 1 DTMUV was carried out in 4-week-old Cherry Valley ducks, as described previously [10]. This specific age group was chosen for pathogenesis assessment due to its high susceptibility to DTMUV infection [10]. In brief, DTMUV-negative ducks (*n* = 35) were inoculated with 10^5^ ELD_50_/ml of cluster 1 DTMUV (DK/TH/CU-DTMUV2007) through the intranasal (0.5 ml) and intramuscular (0.5 ml) routes. An additional group of 35 ducks was mock inoculated with allantoic fluid from specific pathogen free duck eggs in the same fashion to serve as the noninfected control group. These ducks were acquired from a private research farm operating under high biosecurity standards and were verified to be free from common duck viruses, including DTMUV, by virus-specific RT-PCR/PCR and serological assays [6, 17–20]. To determine the transmission of cluster 1 DTMUV, five naïve ducks were introduced to DTMUV-inoculated group 1 day post-inoculation (dpi) to allow direct contact. Clinical signs and body weights were observed daily throughout the 21-day period. Additionally, oropharyngeal (OP) and cloacal (CL) swabs were taken from each duck on days 1–7, 9, 11, 13, 15, 17, 19, and 21 dpi to examine viral shedding. Five ducks from each group were subjected to blood collection for viremia determination and serum neutralization (SN) testing. Subsequently, these ducks were humanely euthanized at 1, 3, 5, 7, 9, 14, and 21 dpi for gross examination. Various tissue samples, including the brain, spleen, bursa, thymus, heart, pancreas, liver, sciatic nerve, lung, trachea, proventriculus, gizzard, and intestine, were collected. These samples were then either frozen at −80°C for viral load measurement or preserved in 10% neutral buffered formalin for subsequent histopathological and immunohistochemical (IHC) analyses. The animal experiment was conducted in the ABSL-2 containment facility at Chulalongkorn University Laboratory Animal Center (CULAC), and it was carried out with the approval of Chulalongkorn University Animal Care and Use Committee (approval number 2073001).

To compare the pathogenesis of cluster 1 DTMUV with cluster 2 DTMUV, the cluster 2.1 DTMUV pathogenesis data was obtained from our previous study, which was performed in the same way as a cluster 1 DTMUV described in this study [10].

### 2.3. Histopathological and Immunohistochemical Examinations

To determine the histopathological lesions, tissue samples from the brain, spleen, bursa, thymus, heart, pancreas, and liver were first preserved in 10% neutral buffered formalin solution and then embedded in paraffin. Four-micrometer-thick sections were subsequently cut and subjected to staining with hematoxylin and eosin (H&E) following standard histopathological protocols. The histopathological score of lymphoid organs, brain, and other tissues was determined based on the criteria described previously [10]. Briefly, the sections of lymphoid organs were assessed and assigned scores based on the following criteria: 0 for no lesion, 1 for ≤30% depletion, 2 for 30%–60% depletion, and 3 for ≥60% depletion. Brain sections were evaluated using the following criteria: 0 for no lesion, 1 for mild lesions characterized by mild perivascular cuffing consisting of 2 and 3 layers of mononuclear cells and focal gliosis, 2 for moderate lesions involving moderate perivascular cuffing comprising 4–6 layers of mononuclear cells and multifocal gliosis, and 3 for severe lesions marked by severe perivascular cuffing having >6 layers of mononuclear cells and diffuse gliosis. Other tissue sections were scored based on the criteria of 0 for no lesion, 1 for mild mononuclear cell infiltration, 2 for moderate congestion with moderate mononuclear cell infiltration, and 3 for severe congestion and necrosis accompanied by severe mononuclear cell infiltration.

Additionally, the presence of antigens specific to flaviviruses in various tissues, including the brain, spleen, thymus, bursa, heart, pancreas, sciatic nerve, lung, trachea, proventriculus, gizzard, and intestine, were assessed through immunohistochemical staining, as described previously [10]. Briefly, the tissue sections were deparaffinized, rehydrated, and subjected to pretreatment in citrate buffer (pH 6.0) using a microwave oven. To inhibit endogenous peroxidase activity, the sections were incubated with a 3% hydrogen peroxide solution (H_2_O_2_) at room temperature for 10 min. Furthermore, the sections were blocked with 2% BSA for 30 min at 37°C. Subsequently, the primary antibody, a mouse monoclonal antibody specific to the Flavivirus antigen group, clone D1-4G2-4-15 (EMD Millipore Corporation, CA, USA), was applied and incubated overnight at 4°C. Following three washes in PBS, the sections were subjected to incubation with the secondary antibody using a polymer system (Dako REALTM EnvisionTM/HRP, Rabbit/Mouse, Dako, Denmark) at room temperature for 45 min. Subsequently, the substrate, 3,3'-diamino-benzidine tetrahydrochloride (DAB), was added. Hematoxylin was used for counterstaining, and the sections were subsequently dehydrated, mounted with permount medium, and examined under a light microscope. Each test included both positive and negative controls.

### 2.4. Reverse Transcription-Quantitative Real-Time PCR (RT-qPCR)

DTMUV loads in serum, tissue, and swab samples were quantified by DTMUV E-specific RT-qPCR, as previously described [10]. Briefly, viral RNA was extracted from serum, tissue, and swab samples using QIAamp Viral RNA Mini Kit (Qiagen®, Hilden, Germany) and RNeasy Mini Kit (Qiagen®, Hilden, Germany), respectively, according to the manufacturer's instructions. The cDNA was converted from 250 ng of total RNA by random hexamers and the Improm-II reverse transcription system (Promega, Wisconsin, USA), and subsequently served as a template for qPCR using TaqMan™ Fast Advanced Master Mix (Applied Biosystems, TX, USA). To assess DTMUV loads in the samples, a standard curve was established using a recombinant plasmid containing the DTMUV E gene. The absolute quantification of DTMUV in the samples was normalized per 250 ng of total RNA. Both samples and standards were examined in triplicate.

### 2.5. Serum Neutralization (SN) Test

To detect the presence of cluster 1 DTMUV-specific antibodies in serum samples, SN test was conducted using baby hamster kidney (BHK-21) cells as previously described [10, 17]. In brief, triplicate serial twofold dilutions of heat-inactivated sera were incubated for 1 hr at 37°C with 100 TCID_50_ of cluster 1 DTMUV (DK/TH/CU-DTMUV2007). After incubation, the virus–serum mixture was introduced into a 96-well plate containing BHK-21 cells. These cells were incubated at 37°C, and the development of cytopathic effects (CPE) was monitored daily for 5 days. The controls included reference DTMUV seropositive and negative sera, uninfected BHK-21 cells, and a back titration of used virus. The titers of cluster 1 DTMUV-specific neutralizing antibodies were expressed based on the reciprocal of the highest serum dilution capable of inhibiting CPE.

To evaluate the antigenic relationship between cluster 1 and 2.1 DTMUVs, a cross-neutralization test was performed, as described above using cluster 1 or 2.1 DTMUV and their respective antisera. Cluster 2.1 DTMUV-specific antisera were obtained from our previous study [10]. The antigenic relatedness (*R*) values were calculated using the Archetti and Horsfall's [21] equation, and the antigenic relationship between these two clusters was assessed based on the *R*-value [13, 21, 22]. An *R*-value between 24% and 49% represents a four- to eight-fold difference, and *R*-value ≤ 24% indicates a greater than eightfold difference [13, 22].

### 2.6. Statistical Analysis

Data were presented as the mean ± standard deviation (SD). A two-tailed, unpaired Student's *t*-test was applied to compare body weights between the DTMUV-inoculated and noninfected control groups, as well as to assess differences in virus shedding, viral loads in serum and tissues, and SN antibody titers between cluster 1 and 2.1 DTMUV-inoculated groups. Gross and histopathological lesion scores differences between cluster 1 and 2.1 DTMUV-inoculated groups were evaluated using a nonparametric Mann–Whitney *U*-test. The statistical analyses were performed with GraphPad Prism 6.0 software (GraphPad Software Inc., La Jolla, CA, USA). Any *P* < 0.05 was considered to indicate statistical significance.

## 3. Results

### 3.1. Clinical Manifestation

**Figure 1:**
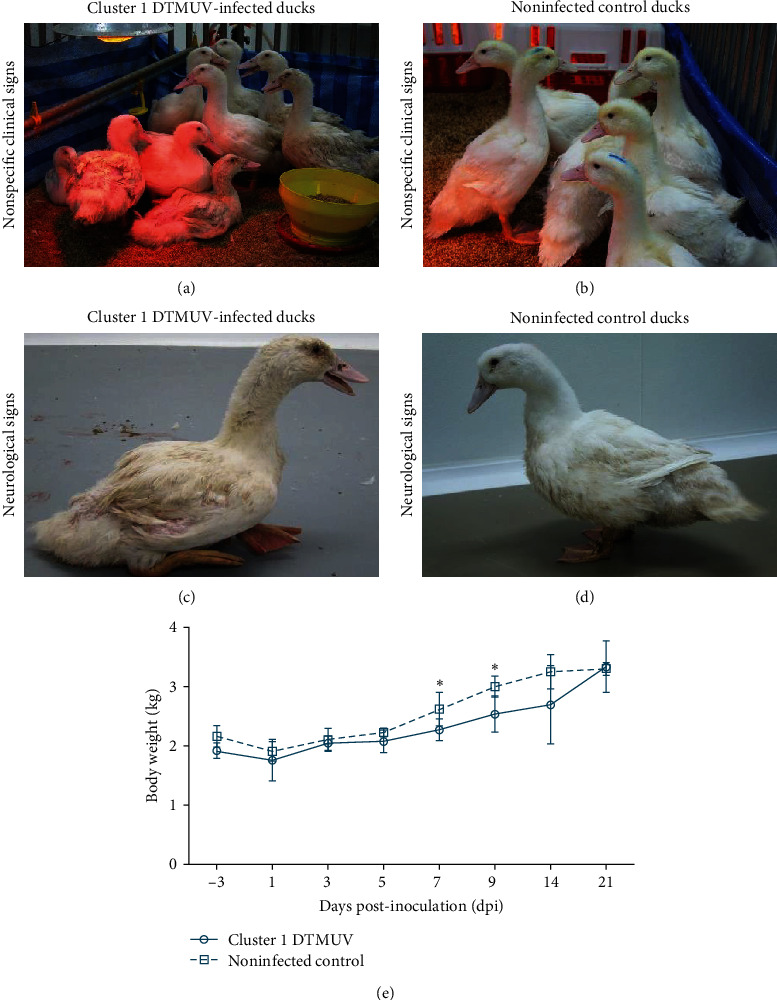
Clinical manifestation of 4-week-old ducks inoculated with cluster 1 DTMUV. (a–d) DTMUV-inoculated ducks showed depression, loss of appetite, and severe neurological signs, including ataxia, reluctance to walk, and paralysis (a, c), while ducks in noninfected control group remained healthy throughout the observation period (b, d). (e) Mean body weights of 4-week-old ducks inoculated with cluster 1 DTMUV compared to noninfected control ducks. Each data point represents the mean body weights ± standard deviation (SD) of five ducks. Asterisks ( ^*∗*^) indicate statistically significant differences between DTMUV inoculated and noninfected control groups (*P* < 0.05, two-tailed Student's unpair *t*-test).

Most ducks inoculated with cluster 1 DTMUV (14/35) displayed depression and loss of appetite as early as 2 dpi (Figures 1(a) and 1(b)). These ducks also exhibited moderate to severe neurological signs, including ataxia, reluctance to walk, and paralysis from 2 to 14 dpi, and continued to show mild neurological signs until the end of observation period (21 dpi) (Figures 1(c) and 1d)). DTMUV-inoculated ducks began to significantly lose weight at 7 dpi and recovered by 21 dpi (Figure 1(e); Table 1). While the morbidity rate of cluster 1 DTMUV was relatively high (40%), none of the inoculated ducks died during the 21-day observation period. Mild to moderate neurological signs were also detected in most contact ducks from 1 to 16 days postcontact (dpc) with 100% (5/5) morbidity; however, no contact ducks died during the observation period. It is noted that these contact ducks were confirmed DTMUV infection by RT-qPCR positive on OP (10^1.4^−10^1.8^ copies) and CL (10^2.9^−10^4.4^ copies) swabs at 4 and 9 dpc. This finding indicated that, like cluster 2.1 DTMUV, cluster 1 DTMUV transmitted efficiently among ducks. Ducks in the noninfected control group remained healthy throughout the 21-day observation period. Compared with a previously reported cluster 2.1 DTMUV [10], cluster 1 DTMUV exhibited slower body weight loss, milder neurological signs, and lower morbidity and mortality rates in 4-week-old ducks (Table 1). Taken together, cluster 1 DTMUV generally induced less severe disease in 4-week-old ducks than cluster 2.1 DTMUV in terms of body weight loss, severity of symptoms, morbidity, and mortality rates.

### 3.2. Gross and Histopathological Findings

**Figure 2:**
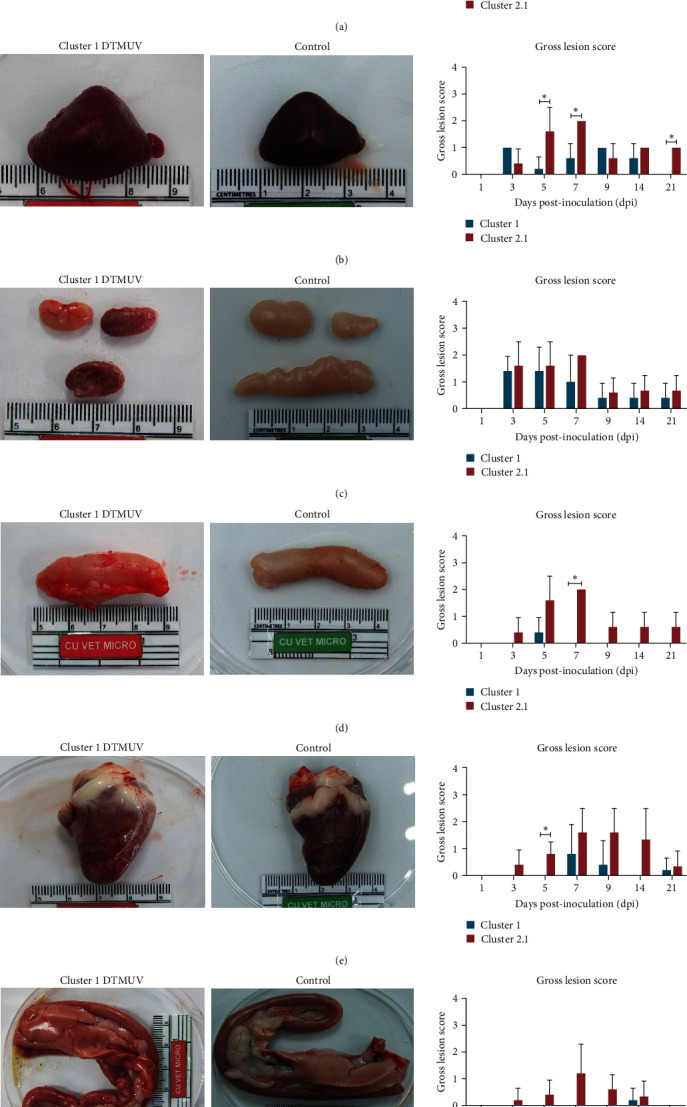
Gross lesions and scores of cluster 1 DTMUV inoculated ducks. (a) Brain: severe congestion and edema. (b) Spleen: swelling and pallor color. (c) Thymus: diffuse petechial hemorrhage and swollen. (d) Bursa: swollen. (e) Heart: petechial hemorrhage in myocardium. (f) Pancreas: multiple petechial hemorrhage and congestion. Bar charts show mean gross lesion scores of cluster 1 DTMUV compared to cluster 2.1 DTMUV. It should be noted that gross lesion scores of cluster 2.1 DTMUV were retrieved from our previous study [10]. Each data point represents the mean gross lesion scores ± standard deviation (SD) of five ducks. Asterisks ( ^*∗*^) indicate statistically significant differences between cluster 1 and 2.1 DTMUV infected groups (*P* < 0.05), a nonparametric Mann–Whitney *U*-test.

The most prominent gross lesions in cluster 1 DTMUV-inoculated ducks were cerebral edema and meningeal congestion and hemorrhages in myocardium and pancreas (Figures 2(a), 2(e), and 2(f)). The major gross lesions of immune organs were spleen enlargement, diffuse petechial hemorrhage and swelling in thymus, and bursa swelling (Figure 2(b)–2(d)). Notably, cluster 1 DTMUV had significantly lower mean gross lesion scores on spleen, bursa, and heart than a previously reported cluster 2.1 DTMUV (Figure 2). No gross lesions were observed in tissues from any of the noninfected control ducks. Overall, the severity of the gross lesions in cluster 1 DTMUV inoculated ducks appeared to be lower than those observed for a previously reported cluster 2.1 DTMUV.

**Figure 3:**
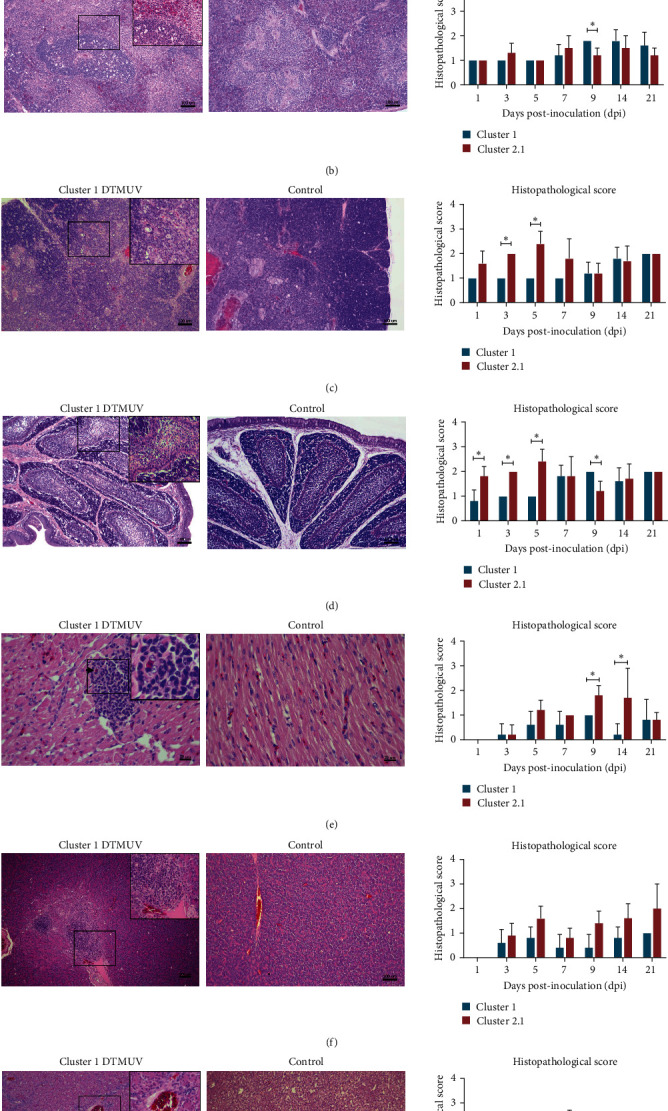
Histopathological lesions and scores of cluster 1 DTMUV inoculated ducks. (a) Brain: severe nonsuppurative perivascular cuffing (white arrows), multifocal gliosis (black arrows), and dark neurons (blue arrows). (b) Spleen: severe germinal lymphoid depletion and congestion. (c) Thymus: severe lymphoid depletion. (d) Bursa: moderate lymphoid depletion. (e) Heart: perivascular cuffing and focal mononuclear cells infiltration in myocardium (arrow indicates blood vessel). (f) Pancreas: severe mononuclear cells infiltration observed mostly at the perivascular area in interlobular fibrous tissue. (g) Liver: severe mononuclear cells infiltration at perivascular area of portal triads. Inserts show higher magnification of the selected square-lesions. Bar charts show mean histopathological scores of cluster 1 DTMUV compared to cluster 2.1 DTMUV. It should be noted that histopathological scores of cluster 2.1 DTMUV were retrieved from our previous study [10]. Each data point represents the mean histopathological scores ± standard deviation (SD) of five ducks. Asterisks ( ^*∗*^) indicate statistically significant differences between cluster 1 and 2.1 DTMUV infected groups (*P* < 0.05), a nonparametric Mann–Whitney *U*-test.

**Figure 4:**
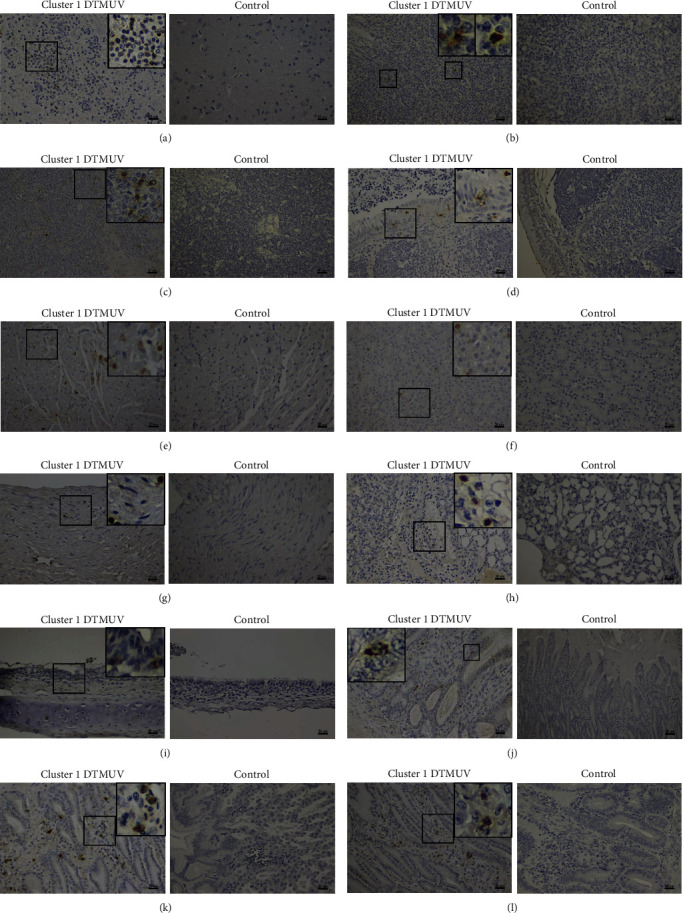
Immunohistochemical (IHC) staining of cluster 1 DTMUV inoculated ducks. The positive DTMUV immunostaining in mononuclear cells in the perivascular area of brain (a). The positive DTMUV immunostaining present in mononuclear cells of spleen (b), thymus (c), bursa (d), heart (e), pancreas (f), nerve (g), lung (h), trachea (i), gizzard (j), proventriculus (k), and intestine (l). Inserts show higher magnification of the selected square-lesions.

**Figure 5:**
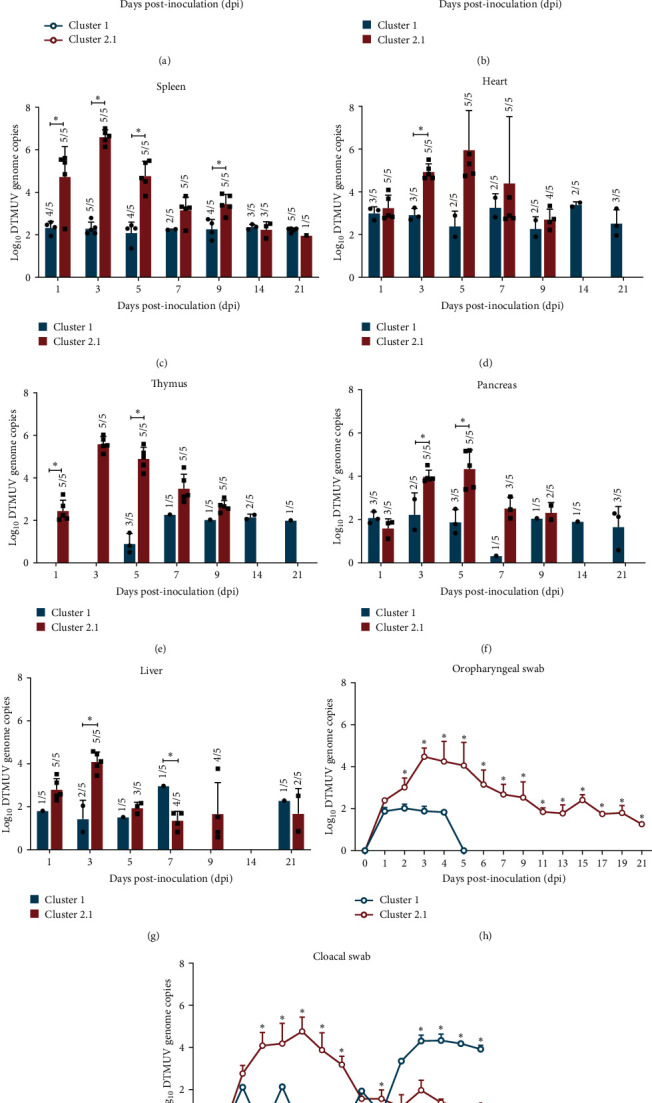
Viremia, viral dissemination, and shedding of cluster 1 DTMUV inoculated ducks. Levels of viremia and viral loads in tissues and swabs were evaluated by RT-qPCR. (a) Viremia levels were expressed as log_10_ DTMUV genome copy number per 50 ng total RNA. Levels of DTMUV RNA in brain (b), spleen (c), heart (d), thymus (e), pancreas (f), liver (g), oropharyngeal (h), and cloacal (i) swabs were expressed as log_10_ DTMUV genome copy number per 250 ng total RNA. It should be noted that all viral load data of cluster 2.1 DTMUV inoculated group were retrieved from a previous study [10]. Each data point represents the mean ± standard deviation (SD). Asterisks ( ^*∗*^) represent statistically significant difference between cluster 1 and 2.1 DTMUV inoculated groups at the indicated time point (*P* < 0.05, two-tailed Student's unpair *t*-test).

The significant histopathological findings in cluster 1 DTMUV inoculated ducks were congestion, perivascular cuffing with mononuclear cells, and necrosis of lymphoid cells in various organs. In the brain, the main histopathological lesion was multifocal gliosis, perivascular cuffing with mononuclear cells, and mild to moderate nonsuppurative encephalitis (Figure 3(a)). In lymphoid tissues, severe lymphoid depletion, lymphocytic death, and hemorrhage were observed in spleen, thymus, and bursa as early as 1 dpi (Figure 3(b)–3(d)). In addition, nonsuppurative perivascular cuffing by mononuclear cell infiltration was mostly observed at perivascular area of various organs, including heart, pancreas, and liver (Figure 3(e)–3(g)). Interestingly, ducks inoculated with cluster 1 DTMUV had significantly lower mean histopathological scores on thymus, bursa, and heart than cluster 2.1 DTMUV inoculated ducks (*P* < 0.05) (Figure 3). No histopathological changes were observed in any of the tested organs from noninfected control ducks. Corresponding to observations on histopathology, IHC analysis revealed the presence of DTMUV antigens in all examined organs of cluster 1 DTMUV inoculated ducks, with different levels of intensity, indicating systemic infection (Figure 4). It is interesting to note that positive immunostaining was more intense and widespread in gastrointestinal tissues than in respiratory tissues of cluster 1 DTMUV inoculated ducks, which was consistent with high and prolonged viral shedding in CL swabs compared to OP swabs (Figures 4 and 5). Similar to cluster 2.1 DTMUV [10], DTMUV antigen was mainly identified in monocytes/macrophages in various tissues of cluster 1 DTMUV inoculated ducks (Figure 4). However, DTMUV immunostaining in cluster 1 DTMUV inoculated group appeared to be weaker and fewer than a previously reported cluster 2.1 DTMUV inoculated group [10] (Figure 4). Altogether, histopathology along with clinical findings and gross pathology suggested that cluster 1 DTMUV was virulent in 4-week-old ducks but was less pathogenic than cluster 2.1 DTMUV.

### 3.3. Viremia, Viral Dissemination, and Shedding

To evaluate the magnitude and kinetic of cluster 1 DTMUV viremia, DTMUV loads in serum samples collected at 1, 3, 5, 7, 9, 14, and 21 dpi were quantified by DTMUV E-specific RT-qPCR. The results demonstrated that viremia in cluster 1 DTMUV inoculated group was first detected at 5 dpi, peaked at 7 dpi, and sustained through 21 dpi (Figure 5(a)). Compared with a previously reported cluster 2.1 DTMUV [10], cluster 1 DTMUV induced delayed and lower levels of viremia (Figure 5(a)), which correlated with reduced disease severity in cluster 1 DTMUV inoculated ducks. No DTMUV RNA was detected in serum samples from any of the noninfected control ducks.

To investigate the tissue dissemination of cluster 1 DTMUV, tissue samples, including brain, spleen, heart, thymus, pancreas, and liver, collected at 1, 3, 5, 7, 9, 14, and 21 dpi were assessed for DTMUV RNA levels. In contrast to delayed serum viremia, DTMUV RNA could be detected in almost all tested tissues of ducks inoculated with cluster 1 DTMUV as early as 1 dpi, suggestive of a rapid systemic spread of cluster 1 DTMUV (Figure 5(b)–5(g)). This observation indicated that the rapid tissue dissemination of cluster 1 DTMUV may occur primarily through cell-associated viremia rather than via cell-free viremia. Although viral loads in all tested organs of cluster 1 DTMUV inoculated ducks reached the maximum levels during 3–7 dpi and mostly sustained through 21 dpi, the levels of viral load in tissues of these ducks were most significantly lower than those observed for cluster 2.1 DTMUV (Figure 5(b)–5(g)). These findings suggested that low viral load levels in visceral organs may be the main cause of decreased disease severity in cluster 1 DTMUV inoculated ducks. It should be noted that, unlike cluster 2.1 DTMUV, the highest viral loads of cluster 1 DTMUV inoculated group were detected in brain (Figure 5(b)–5(g)), indicating that brain might be the major target organ of cluster 1 DTMUV. No DTMUV RNA was observed in any of the noninfected control ducks. Taken together, these findings along with the viremia level suggested that reduced disease severity in cluster 1 DTMUV inoculated ducks might be associated with lower viral load levels both in blood and tissues of cluster 1 DTMUV inoculated ducks compared to cluster 2.1 DTMUV inoculated ducks.

Analysis of cluster 1 DTMUV shedding pattern demonstrated that most ducks inoculated with cluster 1 DTMUV shed the virus in OP swabs as early as 1 dpi; however, the duration of shedding lasted only for 4 days with low-level viral shedding (Figure 5(h)). In contrast to OP shedding, although intermittent viral shedding on CL swabs was observed in some ducks inoculated with cluster 1 DTMUV during the early phase of infection (1–9 dpi), all ducks consistently shed the virus in CL swabs from 13 to 21 dpi, with relatively high shedding level (Figure 5(i)). No DTMUV shedding was found in any of the noninfected control ducks. Overall, cluster 1 DTMUV inoculated ducks generally shed the virus in CL swabs for a longer period and at a higher level than those in OP swabs. This result together with strong and widespread DTMUV immunostaining in gastrointestinal tissues indicated that cluster 1 DTMUV might be spread mainly through the fecal-oral route rather than by the airborne route. Notably, ducks inoculated with cluster 1 DTMUV exhibited significantly lower shedding levels and shorter shedding duration compared to cluster 2.1 DTMUV (*P* < 0.05) (Figures 5(h) and 5(i)). Collectively, these findings, in conjunction with the viral load levels in blood and tissues, revealed that cluster 1 DTMUV replicated less efficiently than cluster 2.1 DTMUV, which might lead to the milder pathogenicity of cluster 1 DTMUV.

### 3.4. Neutralizing Antibody Response

**Figure 6:**
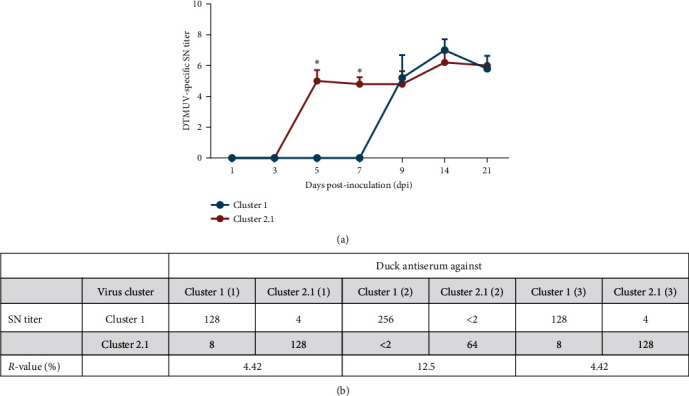
Neutralizing antibody response in ducks inoculated with cluster 1 DTMUV (a) and cross-neutralization activity between cluster 1 and 2.1 DTMUVs (b). All serum neutralization (SN) titer data of cluster 2.1 DTMUV inoculated group were retrieved from a previous study [10]. The antigenic relatedness (*R*) values were calculated using the Archetti and Horsfall's [21] formula and the antigenic relationship between these two clusters was evaluated based on the *R* values. An *R*-value between 24% and 49% represents a four- to eight-fold difference, and *R*-value ≤ 24% indicates a greater than eightfold difference [13, 22]. Asterisks ( ^*∗*^) represent statistically significant difference between cluster 1 and 2.1 DTMUV inoculated groups at the indicated time point (*P* < 0.05, two-tailed Student's unpair *t*-test).

To evaluate the magnitude and kinetic of neutralizing antibody response in ducks inoculated with cluster 1 DTMUV, the titer of cluster 1 DTMUV-specific neutralizing antibodies was assessed by SN test. Following cluster 1 DTMUV inoculation, neutralizing antibodies against cluster 1 DTMUV were first detected at 9 dpi in all ducks, peaked at 14 dpi, and then began to decline but remained detectable at a high level until the end of experiment (Figure 6(a)). Compared with a previously reported cluster 2.1 DTMUV [10], cluster 1 DTMUV induced delayed neutralizing antibody response (Figure 6(a)). However, titers of neutralizing antibodies observed in ducks inoculated with cluster 1 DTMUV were generally comparable to those reported in cluster 2.1 DTMUV (Figure 6(a)). Altogether, these results demonstrated delayed neutralizing antibody response following cluster 1 DTMUV infection in ducks.

To investigate the antigenic relatedness between cluster 1 and 2.1 DTMUVs, a cross-neutralization test was conducted using cluster 1 or 2.1 DTMUVs and their respective antisera. The cross-neutralization results demonstrated partial cross-neutralizations between cluster 1 and 2.1 DTMUVs in some serum samples; however, marked differences in SN titers were observed between homologous and heterologous viruses for all serum samples (Figure 6(b)). Notably, significant antigenic differences were found between cluster 1 and 2.1 DTMUVs (*R* values < 24%) (Figure 6(b)). Collectively, these results revealed that cluster 1 DTMUV displayed not only virulence difference but also antigenic variation compared to cluster 2.1 DTMUV.

## 4. Discussion

The viral strain/genotype is believed to be one of the most important factors affecting the virulence and pathogenicity of DTMUV [12–14]. Most of the DTMUV pathogenesis evaluation has mainly focused on cluster 2, while information on the pathogenesis of other DTMUV clusters, particularly cluster 1, is limited. In this study, the pathogenesis of cluster 1 DTMUV was investigated in 4-week-old ducks and compared to our previously reported cluster 2.1 DTMUV [10]. Our results revealed that cluster 1 DTMUV caused less severe clinical disease compared to cluster 2.1 DTMUV. Concordantly, we also demonstrated that cluster 1 DTMUV replicated less efficiently both in blood and tissues and had overall lower pathogenicity in 4-week-old ducks than cluster 2.1 DTMUV. These findings, in conjunction with the previous evidence [12, 13, 23], support the association between DTMUV genotype and disease severity/pathogenicity. It is interesting to note that, besides pathogenicity difference, significant antigenic variation was found between cluster 1 and 2.1 DTMUVs. Collectively, our data suggested distinct pathogenicity and antigenicity between these 2 DTMUV genotypes. To the best of our knowledge, this is the first study reporting the pathogenesis of cluster 1 Thai DTMUV in ducks and providing the evidence that cluster 1 DTMUV is prone to be less pathogenic than cluster 2.1 DTMUV.

The clinical and pathological analyses demonstrated that cluster 1 DTMUV induced typical clinical features of DTMUV infection in 4-week-old ducks; however, differences in the disease severity and pathogenicity were observed when compared with cluster 2.1 DTMUV [10]. Notably, cluster 1 DTMUV induced milder clinical signs and lesions and had lower morbidity and mortality rates than cluster 2.1 DTMUV in 4-week-old ducks, which clearly indicate that cluster 1 DTMUV was less pathogenic to 4-week-old ducks than cluster 2.1 DTMUV. These observations are in line with the previous studies reporting that cluster 1 Malaysian DTMUV appeared to be less virulent than cluster 2 DTMUV [3, 10, 12]. As reported in cluster 1 Malaysian DTMUV [3] and in the natural infection of cluster 1 DTMUV in Thailand [15], pathological lesions caused by cluster 1 Thai DTMUV were mainly observed in the brain, correlating with severe neurological signs presenting in the inoculated ducks. Apart from brain, cluster 1 Thai DTMUV could also induce pathological changes with positive DTMUV immunostaining in multiple organs of inoculated ducks, indicating systemic infection of cluster 1 DTMUV. Similar to cluster 2.1 DTMUV [10], DTMUV antigen was mainly detected in monocytes/macrophages in various tissues of cluster 1 DTMUV inoculated ducks, supporting a role of monocytes/macrophages tropism in DTMUV pathogenicity [24]. However, consistent with severity of clinical signs and lesions, only sporadic DTMUV immunostaining was found in cluster 1 DTMUV inoculated ducks when compared to ducks inoculated with cluster 2.1 DTMUV [10]. This indicates a decreased replication ability of cluster 1 DTMUV in tissues of inoculated ducks, which possibly leads to milder disease compared with cluster 2.1 DTMUV. Supporting this notion, several previous studies reported the association between the degree of pathogenicity and the level of viral replication capacity *in vivo* [25, 26]. In concordance with a previous observation of cluster 2.1 DTMUV [10], cluster 1 DTMUV contact ducks showed higher morbidity than the inoculated ducks. This may be related to prolonged viral shedding from cloaca of inoculated ducks, which possibly leads to reinfection in contact ducks. Altogether, clinical and pathological findings suggested that cluster 1 DTMUV induced milder pathogenicity compared to cluster 2.1 DTMUV.

Like cluster 2 DTMUV [9–11], DTMUV RNA could also be identified in various organs of ducks inoculated with cluster 1 DTMUV as early as 1 dpi, suggesting the rapid systemic spread and broad tissue tropism of cluster 1 DTMUV in ducks. However, cluster 1 DTMUV displayed a markedly reduced ability to replicate both in tissues and blood compared to cluster 2.1 DTMUV, potentially contributing to the milder pathogenicity than cluster 2.1 DTMUV. These findings, in conjunction with the previous observations [12, 25–27], suggest that the genotype-specific clinical outcomes are likely linked to viral replication capacity. Interestingly, whereas both cluster 1 and 2.1 DTMUVs were able to induce rapid systemic infection, cluster 1 DTMUV caused delayed serum viremia compared to cluster 2.1 DTMUV. A possible explanation for this finding is that cluster 1 DTMUV may invade peripheral organs mainly via cell-associated viremia rather than through cell-free viremia. Supporting this speculation, a previous study showed that DTMUV could infect and replicate efficiently both in monocytes/macrophages and lymphocytes obtained from duck peripheral blood mononuclear cells [24]. In addition, our previous study demonstrated that cluster 1 DTMUV was able to infect and replicate in duck monocytes/macrophages (unpublished data). It is interesting to note that the highest viral loads were detected in brain of ducks inoculated with cluster 1 DTMUV rather than in spleen as reported in cluster 2.1 DTMUV [10]. This finding along with strong DTMUV immunostaining in brain supports the notion that brain is likely to be the main target organ of cluster 1 DTMUV in 4-week-old ducks. We also found that cluster 1 DTMUV persisted in most tissues of infected ducks until the end of observation period. This might have resulted from delayed production of neutralizing antibodies in ducks inoculated with cluster 1 DTMUV, which potentially leads to delayed viral clearance in the tissues. Supporting this speculation, our previous studies showed that the induction of high neutralizing antibody titers early in the course of cluster 2.1 DTMUV infection was associated with the reduction of viral loads in various tissues of the infected ducks [7, 10]. However, the exact mechanism contributing to this event requires further investigation.

Dynamics of viral shedding following cluster 1 DTMUV infection were monitored in OP and CL swabs. Our results revealed a marked difference in shedding pattern between OP and CL swabs of ducks inoculated with cluster 1 DTMUV. In contrast to cluster 2.1 DTMUV [10], CL shedding was generally higher and longer than OP shedding for cluster 1 DTMUV, suggesting that CL swabs might be more appropriate than OP swabs for cluster 1 DTMUV detection. However, short shedding in OP swabs and intermittent shedding in CL swabs appear to limit the utility of these specimens for detecting cluster 1 DTMUV as compared to tissues, especially the brain. These findings along with a higher number of DTMUV antigen-positive cells in gastrointestinal tissues compared to respiratory tissues indicated that cluster 1 DTMUV transmission was more likely via the fecal-oral route rather than through the airborne route. Concordantly, high level of DTMUV RNA (10^2.9^–10^4.4^ copies) could be detected mostly in CL swabs from contact ducks showing typical signs of DTMUV infection, suggesting the potential transmission of cluster 1 DTMUV mainly through the fecal-oral route. Supporting this notion, a recent study showed that, unlike cluster 2 DTMUV [9, 10], cluster 3 DTMUV could not be transmitted via direct contact, indicating the potential association between the main route of transmission and DTMUV genotypes [28]. Interestingly, shorter shedding duration with lower viral loads could be observed in ducks inoculated with cluster 1 DTMUV compared with a previously reported cluster 2.1 DTMUV [10]. This might be related to a reduced viral replication capacity of this cluster in ducks compared to cluster 2.1 DTMUV. Collectively, cluster 1 DTMUV exhibited a marked decrease in viral replication and shedding abilities compared to cluster 2.1 DTMUV, thus providing a potential explanation as to why current predominant circulation is mostly attributed to cluster 2.1 DTMUV [2, 23].

Compared with a previously reported cluster 2.1 DTMUV [10], delayed seroconversion was observed in ducks inoculated with cluster 1 DTMUV. This difference may be attributed to the distinct viral replication capacity of each cluster in ducks. It should be noted that although the severity of disease gradually declined over the course of cluster 1 DTMUV infection, no obvious correlation was observed between neutralizing antibody titers and viral loads in blood and tissues, which contrasted with those observed in cluster 2.1 DTMUV [10, 29]. This suggested that other immunological mechanisms, particularly cellular immune response, may be involved in the control of cluster 1 DTMUV infection in ducks, which warrants further investigation. Interestingly, our results demonstrated a marked antigenic difference between cluster 1 and 2.1 DTMUV. This difference may be attributed to amino acid substitutions in the E protein. Supporting this notion, our previous study showed that amino acid changes in cluster 1 DTMUV were mainly observed in the E protein, especially in the domain III (DIII), which is known to be responsible for the induction of virus-neutralizing antibodies [15]. Consistent with this finding, a previous study revealed that a significant antigenic difference was also found between cluster 2 and 3 DTMUVs [13]. Therefore, our data together with this previous finding suggests the potential relationship between neutralizing activity and DTMUV clusters/genotypes. However, whether these antigenic differences affect the cross-protection among DTMUV genotypes should be urgently investigated.

Our results collectively demonstrated that the pathogenicity of cluster 1 DTMUV was lower than those of cluster 2.1 DTMUV as evidenced by lower morbidity and mortality rates, milder pathological changes, and reduced viral loads. The difference in the pathogenicity and disease severity between these two clusters may be partly associated with the variation in inducing host immune responses, particularly cellular immune responses. A previous study showed that highly virulent DTMUV elicited stronger cellular immune response than low virulent DTMUV, whereas both of them induced comparable levels of neutralizing antibodies [14]. Additional studies are required to clarify the role of host immune responses on the pathogenicity and virulence differences between cluster 1 and 2.1 DTMUVs. Furthermore, the pathogenicity variation between cluster 1 and 2.1 DTMUVs could be resulted from functional differences in the viral genome or proteins. Our previous study revealed a marked genetic divergence between cluster 1 and 2.1 DTMUVs, most of which were located in NS5, NS1, and E genes [15]. Several studies have shown that minor changes in the genome of DTMUV, especially the E and NS5 genes, have a large impact on pathogenicity and disease severity [30–34]. However, the molecular determinants responsible for the pathogenicity difference observed between cluster 1 and 2.1 DTMUVs remain unknown and require further investigation.

In conclusion, our data collectively indicated that cluster 1 DTMUV was less pathogenic and virulent than a previously reported cluster 2.1 DTMUV in 4-week-old ducks. Overall, our findings suggest differences in the antigenicity and pathogenicity between DTMUV genotypes in ducks, highlighting the potential association between DTMUV genotype and pathogenicity/disease severity. This study enhances our understanding of the pathogenesis of DTMUV infection in ducks and provides useful information for the design and development of effective DTMUV vaccines.

## Figures and Tables

**Table 1:** Comparative clinical manifestation of cluster 1 and 2.1 DTMUV infections in 4-week-old ducks.

DTMUV cluster	Neurological signs (no. of duck/severity)	Morbidity rate (%)	Mortality rate (%)	Body weight loss (duration; day)
1	14/++^a^	40	0	7–14
2.1	18/+++	51.42	22.86	3–21

*Note*: ^a^The severity of neurological signs is shown as + (mild), ++ (moderate), and +++ (severe). Clinical data of cluster 2.1 DTMUV were retrieved from our previous study [10].

## Data Availability

The data that support the findings of this study are available from the corresponding author upon reasonable request.
